# Special Issue on “Smart City and Smart Infrastructure”

**DOI:** 10.3390/s21217064

**Published:** 2021-10-25

**Authors:** Sung-Han Sim, Jong-Jae Lee

**Affiliations:** 1 School of Civil, Architectural Engineering and Landscape Architecture, Sungkyunkwan University, Suwon 16419, Korea; ssim@skku.edu; 2 Department of Civil and Environmental Engineering, Sejong University, Seoul 05006, Korea

Recent developments in sensor technologies and data-driven approaches have been recognized as the main enablers of smart cities. Transforming a traditional city into a smart city requires a wide variety of unprecedented challenges in various fields, including smart infrastructure monitoring, public safety, waste management, smart lighting solutions, and smart transportation [1]. Issues related to smart cities can be addressed effectively by the appropriate use of sensors and state-of-the-art data processing schemes for better realization of the smart city.

Sensors and sensor networks are the most important components of a smart city, acquiring various types of data containing useful information. Transducers that can measure structural responses, such as acceleration, displacement, and strain, have been commonly used in the health monitoring of smart infrastructure [2,3,4]. Environmental sensors (e.g., thermometers, humidity sensors, anemometers, and light sensors) have also been utilized to account for environmental effects in target applications [5,6]. Position information, which is typically collected by the global position system (GPS), enables understanding the behavior of GPS-equipped entities, such as human beings and vehicles [1]. Wireless smart sensors can significantly reduce the installation and maintenance costs of the monitoring system, in addition to allowing multi-sensor data to be collected from a dense network of sensors [7,8,9]. Furthermore, cameras as sensing devices have been adopted in many studies for smart cities and smart infrastructure [10,11,12]. As such, sensor-based developments have been an essential part of smart city research.

The development of advanced data processing and decision-making algorithms has been another research focus in the fields of smart cities and smart infrastructure. It includes deep learning and computer vision applications [10,13,14,15,16], probabilistic prediction and estimation of structural conditions [17], data processing schemes tailored to sensor developments such as magnetic flux leakage (MFL) [18], and light detection and ranging (LiDAR) [19]. In particular, deep learning in conjunction with computer vision has revolutionized research into smart cities. A typical example is a visual inspection for infrastructure maintenance with the automated acquisition of image information and processing to detect defects such as cracks in concrete structures and pavements, corrosion, and spalling of concrete covers [20]. Indeed, these advanced algorithms are necessary to realize smart cities for the best use of sensor data.

This article reviews the technical papers in the Special Issue of “Smart City and Smart Infrastructure” published in the *Sensors* journal. The Special Issue includes a total of 11 papers that have presented state-of-the-art technologies, ranging from machine learning approaches for smart city applications to sensor-based intelligent monitoring of civil infrastructure. This Special Issue is considered to reflect the most recent trend in smart city-related research.

The most salient features of this Special Issue are the use of data-driven models, such as machine learning and the development of intelligent sensor systems. In particular, it has been shown that image data with machine learning enable efficient data processing and extraction of important, meaningful information in smart city applications. In addition, the development of sensor systems to acquire data containing useful features is another focus of the studies in this Special Issue.

A total of five papers in this issue employ image-based approaches with deep learning for various smart city and infrastructure monitoring applications. Jang et al. [10] developed a railway inspection system that uses a line scan camera and an image-based deep learning model to monitor railway facilities efficiently. The line scan camera captured images of railway facilities while the train moved. The acquired image data are subsequently fed into the deep learning model to detect damage to the railway facilities. Kwak and Lee [13] proposed a depth-estimation method using images and deep learning. This fundamental research provides a range of information from 2D images, which are known to be useful for virtual reality, augmented reality (AR), and autonomous driving. Another study tailored to AR is the semantic segmentation method proposed by Ko and Lee [14]. This method, which consists of a modified dilated residual network, an atrous pyramid pooling module, and back propagation, is designed to have high computational efficiency for AR applications with a frame rate higher than 60 frames per second (fps). Another study on image processing is low-light image enhancement using deep learning [15]. This method is remarkably useful in handling images captured by surveillance cameras at night. The images enhanced by the method allow the extraction of important features. A notable paper in this Special Issue is the image-based approach using a convolutional neural network for the detection of road surface damage [16]. This method features data augmentation tailored to road images and semi-supervised learning to improve the data detection performance.

Data-driven methodologies can also be used with other types of sensor data rather than images. Szeląg et al. [21] applied a data-driven approach to identify the activated sludge bulking in wastewater treatment plants. The results indicated that the method helps to choose the computation methods used in simulating sludge bulking with minimized measurement costs. Qarout et al. [1] used GPS data in probabilistic modeling to understand human behavior in urban areas. The proposed approach, the adaptive input hidden Markov model (AI-HMM), was shown to be capable of grouping different categories of human behavior trends and identifying time-specific anomalies. AI-HMM has a strong potential as an alternative to costly motion imagery and privacy-invading surveillance cameras, which have already been in use. Related research was conducted by Nguyen et al. [22], developing a smartphone-based method using acceleration data for real-time passenger tracking in the London underground tube. In contrast to the study by Qarout et al., GPS is unavailable in an underground environment; therefore, acceleration time series were employed alternatively to track passengers. An interesting aspect of this work is that the proposed method utilizes the fact that the underground tubes run on the designated tracks and, thus, the vibration characteristics and locations are highly correlated. Principal component analysis was selected and trained with the measured acceleration data and location information, achieving an accuracy of up to 18 m in 90% of the total time. Another notable example of data-driven methods was demonstrated by Lee et al. [17], which is a Bayesian prediction model for bridge deflection. The prediction model was trained using long-term deflection data measured during the construction stage of a railway bridge. The finite element (FE) model of the testbed bridge provided estimated deflections in each construction stage, such as concrete pouring, to build a slab on top of the girders, which were constructed prior to the concrete slab. As the prediction model utilizes the estimated deflections from the FE model, it can be viewed as a hybrid method that uses measured data and a numerical model in combination. Kim et al. [23] conducted data-driven research for infrastructure monitoring using long-term measurement data. Structural responses of vibration data and wind speed data were utilized in the buffeting analysis of a cable-stayed bridge.

Studies using advanced sensor systems are also a core topic in this Special Issue. Azad and Kim [18] presented an MFL coil sensor for the nondestructive inspection of stay cables. The optimal parameters for accurate and efficient damage detection were determined numerically and used to fabricate an MFL coil sensor system. The experiment in the laboratory environment was conducted using a steel bar specimen with a defect, which was successfully identified. Furthermore, some of the papers regarding data-driven approaches discussed previously exhibit interesting sensor systems. Lee et al. [17] used a dual-camera system developed for long-term displacement sensing [11]. The computer vision system acquired displacement data with a sampling rate of 1 min, which was used for the prediction model. The railway inspection system by Jang et al. [10] features a line scan camera installed on top of the trains, which produces scanning images of a long railway line. This image sensing system, tailored to the railway, is optimal for this specific problem. Nguyen et al. [22] used smartphones to measure acceleration data and process the measured data in real time. As such, advanced sensing devices combined with intelligent data processing provide a new horizon for next-generation smart city research.

## Data Availability

Not acceptable.
